# Inference of ceftobiprole susceptibility through surrogate testing of ceftaroline

**DOI:** 10.1128/jcm.01706-25

**Published:** 2026-02-24

**Authors:** Helio S. Sader, Rodrigo E. Mendes, David P. Nicolau, Kristie K. Zappas, Mariana Castanheira

**Affiliations:** 1 Element Iowa City (JMI Laboratories) 138461, North Liberty, Iowa, USA; 2 Innoviva Specialty Pharmaceuticals, Waltham, Massachusetts, USA; Cleveland Clinic, Cleveland, Ohio, USA

**Keywords:** ceftobiprole, ceftaroline, *Staphylococcus aureus*, *Streptococcus pneumoniae*, *Escherichia coli*, *Klebsiella pneumoniae*, *Haemophilus influenzae*

## Abstract

Ceftobiprole is an advanced-generation cephalosporin active against methicillin-resistant *Staphylococcus aureus* and other clinically relevant bacteria. Ceftobiprole was recently (April 2024) approved for clinical use in the United States, but susceptibility testing is still not available on commercial automated susceptibility testing systems. We evaluated the accuracy and error rates when using susceptibility to ceftaroline to predict susceptibility to ceftobiprole. A total of 42,363 clinical isolates were collected through the SENTRY Antimicrobial Surveillance Program from 34 US medical centers in 2016–2020 and tested against ceftobiprole and ceftaroline by broth microdilution method. The accuracy of the surrogate test (susceptibility to ceftaroline) was defined as the percentage of isolates susceptible to ceftobiprole among ceftaroline-susceptible isolates. Minor error rate was then defined as the percentage of ceftobiprole-intermediate among ceftaroline-susceptible isolates, and very major error was defined as the percentage of ceftobiprole-resistant isolates among ceftaroline-susceptible isolates. The accuracy of the surrogate test to predict susceptibility to ceftobiprole was >99% for *S. aureus* (99.98%), including methicillin-resistant *S. aureus* (99.95%), *Streptococcus pneumoniae* (99.48%), β-hemolytic streptococci (100.00%), *Escherichia coli* (99.97%), and *Klebsiella pneumoniae* (99.91%). Accuracy was also extremely high for *Haemophilus* spp. (98.86%) and Enterobacterales (98.73%). Minor error rates were <1% for all organism groups evaluated, and very major errors were only observed with *S. pneumoniae* (0.06%), *Haemophilus* spp. (0.31%), Enterobacterales (0.40%), and *K. pneumoniae* (0.03%). In conclusion, the results of the comparative analyses of ceftobiprole and ceftaroline MIC values show that susceptibility to ceftobiprole can be inferred by susceptibility to ceftaroline with very high accuracy.

**IMPORTANCE:**

Ceftobiprole was recently (April 2024) approved for clinical use in the United States but susceptibility testing is still not available on commercial automated susceptibility testing systems and may not be available for some time. In the interim, one possible strategy to assess ceftobiprole susceptibility would be to apply the susceptibility results of ceftaroline as a surrogate marker of susceptibility. This strategy could facilitate rapid clinical use of ceftobiprole in the United States pending the more necessary availability of commercial susceptibility testing devices to direct testing ceftobiprole.

## INTRODUCTION

Ceftobiprole is a fifth-generation intravenous cephalosporin with broad-spectrum activity that includes many clinically relevant gram-positive and gram-negative bacteria, such as *Staphylococcus aureus* (including methicillin-resistant [MRSA] strains), *Streptococcus pneumoniae* (including penicillin nonsusceptible), *Haemophilus influenzae*, β-hemolytic streptococci, non-extended-spectrum β-lactamase (ESBL)–producing Enterobacterales, *Moraxella catarrhalis*, *Enterococcus faecalis*, and *Pseudomonas aeruginosa* (1, 2).

Ceftobiprole was approved by the United States Food and Drug Administration (FDA) in April 2024 for the treatment of adults with *S. aureus* bloodstream infections (bacteremia), including those with right-sided infective endocarditis; adults with acute bacterial skin and skin structure infections; and adult and pediatric patients 3 months to less than 18 years old with community-acquired bacterial pneumonia (https://www.fda.gov/news-events/press-announcements/fda-approves-new-antibiotic-three-different-uses) (3, 4). Furthermore, ceftobiprole has been marketed in Europe and other regions for the treatment of community- and hospital-associated pneumonia for many years (5–7).

Susceptibility testing for ceftobiprole is not currently available on commercial automated susceptibility testing systems used in the United States and may not be available for some time (8). In the interim, one possible strategy to assess ceftobiprole susceptibility would be to apply the susceptibility results of a commonly tested and similar cephalosporin class, such as ceftaroline, as a surrogate marker of susceptibility (9–11). Both ceftobiprole and ceftaroline act by binding tightly to the penicillin-binding proteins (PBPs), including those responsible for β-lactam resistance in staphylococci (PBP2a) and pneumococci (PBP2×). Moreover, mechanisms of acquired resistance to these agents are generally similar (PBP alterations among gram-positive and β-lactamases among Enterobacterales) and adversely affect both compounds (2). In this investigation, we assessed the accuracy and error rates when using susceptibility to ceftaroline as a surrogate test to predict susceptibility to ceftobiprole. These assessments were performed by comparing the MIC results and categorical agreement between these 2 compounds when testing 42,363 clinical isolates from US medical centers.

## MATERIALS AND METHODS

A total of 42,363 clinical bacterial isolates were collected through the SENTRY Antimicrobial Surveillance Program from 34 US medical centers in 2016–2020 and included in this investigation (12). The organism collection included 13,867 *Staphylococcus aureus* (5,906 MRSA and 7,961 methicillin-susceptible [MSSA] isolates), 3,245 *Streptococcus pneumoniae*, 1,768 β-hemolytic streptococci, 2,278 *Haemophilus* spp. (2,248 *Haemophilus influenzae* and 30 *H. parainfluenzae*), and 21,205 Enterobacterales (including 9,927 *Escherichia coli*, 4,054 *Klebsiella pneumoniae*, and 7,224 isolates of other species).

Isolates were tested against ceftobiprole and ceftaroline by broth microdilution methods according to Clinical Laboratory Standards Institute (CLSI) standards (13). Element Iowa City (JMI Laboratories; North Liberty, IA, USA) produced frozen-form 96-well panels. The testing medium was cation-adjusted Mueller-Hinton broth (CAMHB), except for streptococci (CAMHB supplemented with 2.5%–5% lysed horse blood) and *Haemophilus* spp. (*Haemophilus* Test Medium broth). CAMHB was acquired from Becton, Dickinson and Company (BD BBL; catalog no. 212,322; Franklin Lakes, NJ, USA). Laked horse blood was from HemoStat Laboratories (catalog no. LHB/Dl1; Dixon, CA, USA), hematin porcine and β-NAD from Sigma-Aldrich Company (catalog no. H328101G and 43,420, respectively; Saint Louis, MO, USA), and yeast extract from Gibco/Thermo Fisher Scientific (catalog no. 212,750; Waltham, MA, USA). The most recent CLSI M100 breakpoint criteria, which are recognized by the US FDA, were used for ceftaroline, and the US FDA breakpoint criteria were used for ceftobiprole (14, 15) (Table 1).

**TABLE 1:** MIC breakpoints for ceftobiprole (US FDA) and ceftaroline (CLSI)^*e*^

Organism	Interpretative categories and MIC breakpoints (mg/L)						
	Ceftobiprole^*a*^			Ceftaroline^*b*^			
	S	I	R	S	I	SDD	R
*S. aureus* (including MRSA)	≤2	4	≥8	≤1	–^*d*^	2–4	8
*H. influenzae*	≤0.5	–	≥1	≤0.5	–	–	–
*S. pneumoniae* (nonmeningitis)	≤0.5	1	≥2	≤0.5	–	–	–
*Streptococcus pyogenes*/β-hemolytic streptococci**^*c*^**	≤0.5	–	–	≤0.5	–	–	–
Enterobacterales	≤0.5	–	1	≤0.5	1	–	2

^*a*^ US FDA (15).

^*b*^ CLSI M100 document (14).

^*c*^ *Streptococcus pyogenes* per US FDA (15) and β-hemolytic streptococci per CLSI M100 document (14).

^*d*^ “–” indicates that no value is published.

^*e*^ S, susceptible; I, intermediate; R, resistant; SDD, susceptible dose–dependent.

Data analysis followed the general intermethod comparison guidelines found in CLSI document M52 (16, 17). Interpretations for ceftobiprole focused on the use of a single surrogate agent (ceftaroline) to predict susceptibility to ceftobiprole. This surrogacy analysis included scattergram graphs between ceftobiprole and ceftaroline MIC results for each group of organisms and indicated species and then applying the respective current susceptibility interpretive criteria.

The goal of this investigation was to evaluate accuracy and error rates when using susceptibility to ceftaroline to predict susceptibility to ceftobiprole. Accordingly, only the group of isolates susceptible to ceftaroline was used to calculate error rates. The accuracy of the surrogate marker (susceptibility to ceftaroline) was defined as the percentage of isolates susceptible to ceftobiprole among ceftaroline-susceptible isolates. Minor error rate was then defined as the percentage of ceftobiprole-intermediate isolates among ceftaroline-susceptible isolates, and a very major error was defined as the percentage of ceftobiprole-resistant isolates among ceftaroline-susceptible isolates (16, 17).

## RESULTS

The cumulative percentages of isolates from nine pathogen groups inhibited by increasing concentrations of ceftobiprole and ceftaroline are shown in Table 2. Against *S. aureus*, ceftobiprole (MIC_50/90_, 0.5/2 mg/L; 99.7% susceptible) MIC values were generally one doubling dilution higher than the MIC values of ceftaroline (MIC_50/90_, 0.25/1 mg/L; 97.4% susceptible). Both ceftobiprole (MIC_50/90_, 0.5/0.5 mg/L; 100.0% susceptible) and ceftaroline (MIC_50/90_, 0.25/0.25 mg/L; 100.0% susceptible) were highly active against MSSA. When tested against MRSA, ceftobiprole MIC values were generally one doubling dilution higher (MIC_50/90_, 1/2 mg/L), but the susceptibility rate was higher (99.2%) when compared to ceftaroline (MIC_50/90_, 1/1 mg/L; 94.0% susceptible; Table 2). It is important to recognize that the susceptible breakpoint established by the US FDA for ceftobiprole (≤2 mg/L) is one doubling dilution higher than the susceptible breakpoint currently published by CLSI for ceftaroline (≤1 mg/L).

**TABLE 2:** Antimicrobial activity of ceftobiprole and ceftaroline tested against the main organisms and organism groups

Organism/organism group (no. of isolates)	No. of isolates (cumulative %) inhibited at MIC (mg/L) of^*a*^:																MIC_50_	MIC_90_
	≤0.001	0.002	0.004	0.008	0.015	0.03	0.06	0.12	0.25	0.5	1	2	4	8	16	>^*b*^		
*Staphylococcus aureus*																		
Ceftobiprole (13,867)						1 (<0.1)	6 (0.1)	60 (0.5)	2,234 (16.6)	5,968 (59.6)	3,994 (88.4)	1,558 (**99.7**)	46 (100.0)				0.5	2
Ceftaroline (13,867)							70 (0.5)	1,098 (8.4)	6,552 (55.7)	2,907 (76.6)	2,883 (**97.4**)	356 (>99.9)	1 (100.0)				0.25	1
Methicillin-susceptible *Staphylococcus aureus*																		
Ceftobiprole (7,961)						1 (<0.1)	6 (0.1)	58 (0.8)	2,216 (28.7)	5,660 (99.7)	20 (**100.0**)						0.5	0.5
Ceftaroline (7,961)							70 (0.9)	1,092 (14.6)	6,438 (95.5)	3,61 (**100.0**)							0.25	0.25
Methicillin-resistant *Staphylococcus aureus*																		
Ceftobiprole (5,906)							0 (0.0)	2 (<0.1)	18 (0.3)	308 (5.6)	3,974 (72.8)	1,558 (**99.2**)	46 (100.0)				1	2
Ceftaroline (5,906)							0 (0.0)	6 (0.1)	114 (2.0)	2,546 (45.1)	2,883 (**94.0**)	356 (>99.9)	1 (100.0)				1	1
*Streptococcus pneumoniae*																		
Ceftobiprole (3,245)	5 (0.2)	19 (0.7)	88 (3.5)	946 (32.6)	1,074 (65.7)	137 (69.9)	137 (74.1)	176 (79.6)	370 (91.0)	276 (**99.5**)	15 (99.9)	2 (100.0)					0.015	0.25
Ceftaroline (3,245)				1,947 (60.0)	223 (66.9)	248 (74.5)	356 (85.5)	425 (98.6)	38 (99.8)	8 (**100.0**)							≤0.008	0.12
β-hemolytic streptococci																		
Ceftobiprole (1,768)	1 (0.1)	11 (0.7)	35 (2.7)	671 (40.6)	497 (68.7)	535 (99.0)	15 (99.8)	3 (**100.0**)									0.015	0.03
Ceftaroline (1,768)				1,113 (63.0)	626 (98.4)	29 (**100.0**)											≤0.008	0.015
*Haemophilus* spp.																		
Ceftobiprole (2,278)				16 (0.7)	128 (6.3)	866 (44.3)	811 (79.9)	356 (95.6)	71 (**98.7**)	19 (99.5)	6 (99.8)					5 (100.0)	0.06	0.12
Ceftaroline (2,278)			279 (12.2)	922 (52.7)	678 (82.5)	279 (94.7)	76 (98.1)	20 (98.9)	18 (99.7)	1 (**99.8**)						5 (100.0)	0.008	0.03
Enterobacterales																		
Ceftobiprole (21,205)				112 (0.5)	448 (2.6)	11,365 (56.2)	4,156 (75.8)	1,033 (80.7)	524 (**83.2**)	324 (84.7)	207 (85.7)	214 (86.7)	172 (87.5)	68 (87.8)	58 (88.1)	2,524 (100.0)	0.03	>16
Ceftaroline (21,205)							7,299 (34.4)	4,715 (56.7)	2,416 (68.0)	1,549 (**75.4**)	967 (79.9)	402 (81.8)	222 (82.9)	214 (83.9)		3,421 (100.0)	0.12	>8
*Escherichia coli*																		
Ceftobiprole (9,297)				2 (<0.1)	139 (1.5)	5,762 (63.5)	1,526 (79.9)	206 (82.1)	107 (**83.3**)	57 (83.9)	21 (84.1)	9 (84.2)	13 (84.3)	9 (84.4)	12 (84.6)	1,434 (100.0)	0.03	>16
Ceftaroline (9,297)							4,128 (44.4)	1,940 (65.3)	872 (74.6)	361 (**78.5**)	223 (80.9)	101 (82.0)	45 (82.5)	52 (83.1)		1,575 (100.0)	0.12	>8
*Klebsiella pneumoniae*																		
Ceftobiprole (4,054)				2 (<0.1)	110 (2.8)	2,495 (64.3)	591 (78.9)	103 (81.4)	36 (**82.3**)	23 (82.9)	26 (83.5)	32 (84.3)	15 (84.7)	10 (84.9)	11 (85.2)	600 (100.0)	0.03	>16
Ceftaroline (4,054)							1,451 (35.8)	1,198 (65.3)	364 (74.3)	211 (**79.5**)	79 (81.5)	50 (82.7)	38 (83.6)	28 (84.3)		635 (100.0)	0.12	>8

^*a*^ Values in bold indicate percentage susceptible.

^*b*^ Greater than the highest concentration tested.

Similar to *S. aureus*, ceftobiprole MIC values were one doubling dilution higher than ceftaroline when tested against *S. pneumoniae* (MIC_50/90_, 0.015/0.25 mg/L for ceftobiprole and ≤0.008/0.12 mg/L for ceftaroline) and β-hemolytic streptococci (MIC_50/90_, 0.015/0.03 mg/L for ceftobiprole and ≤0.008/0.015 mg/L for ceftaroline; Table 2). Of note, the susceptible breakpoint is ≤0.5 mg/L for both drugs against both organisms. When tested against *Haemophilus* spp., ceftobiprole MIC values (MIC_50/90_, 0.06/0.12 mg/L; 98.7% susceptible) were one to two doubling dilutions higher than ceftaroline (MIC_50/90_, 0.008/0.03 mg/L; 99.8% susceptible; Table 2).

Among 21,205 Enterobacterales isolates tested, ceftobiprole (MIC_50/90_, 0.03/>16 mg/L; 83.2% susceptible) MIC values were generally two doubling dilutions lower than the MIC values of ceftaroline (MIC_50/90_, 0.12/>8 mg/L; 75.4% susceptible). Similar differences between ceftobiprole and ceftaroline were observed for *E. coli* and *K. pneumoniae* in terms of MIC values (MIC_50/90_, 0.03/>16 mg/L for ceftobiprole and 0.12/>8 mg/L for ceftaroline for both organisms) and susceptibility rates (83.3% and 82.3% susceptible to ceftobiprole and 78.5% and 79.5% susceptible to ceftaroline, respectively; Table 2). Thus, susceptibility rates were generally higher for ceftobiprole compared to ceftaroline against Enterobacterales species. Notably, the ceftobiprole-susceptible breakpoint (≤0.25 mg/L) for Enterobacterales is one doubling dilution lower than the ceftaroline-susceptible breakpoint (≤0.5 mg/L).

**Fig 1:**
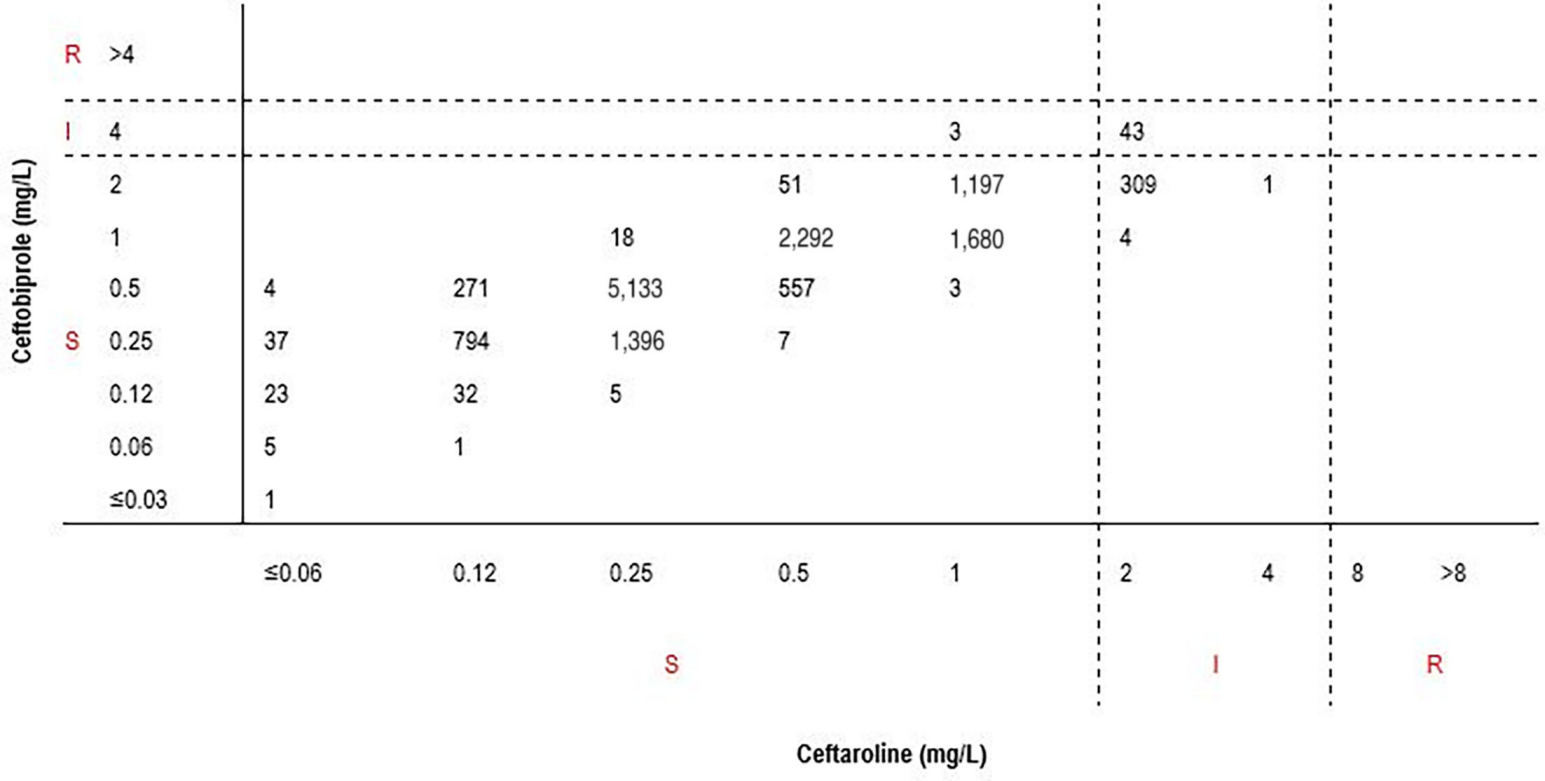
Correlation between ceftobiprole and ceftaroline MIC values for *Staphylococcus aureus* (*n* = 13,867)

Figure 1 shows the correlation between ceftobiprole and ceftaroline MIC values for *S. aureus*. Among 13,867 isolates tested, 13,507 isolates (97.40%) were susceptible, and 43 isolates (0.31%) were intermediate to both ceftobiprole and ceftaroline, indicating a total categorical agreement of 97.71% between these two compounds. Importantly, among 13,510 *S. aureus* isolates susceptible to ceftaroline, 13,507 (99.98%) were also susceptible to ceftobiprole. Therefore, the predictable ceftobiprole susceptibility rate was 99.98% for *S. aureus* isolates susceptible to ceftaroline. Three isolates (0.02%) were susceptible to ceftaroline and intermediate to ceftobiprole, resulting in a minor error rate of 0.02%. There were no very major errors (Table 3). Additionally, 314 of 357 (87.96%) isolates intermediate to ceftaroline were susceptible to ceftobiprole (Fig. 1).

**TABLE 3:** Summary of ceftaroline test result accuracy for predicting ceftobiprole susceptibility using ceftaroline breakpoints established by CLSI and ceftobiprole breakpoints established by the US FDA^*e*^

Pathogen or species group (no. tested/no. ceftaroline-susceptible)	Accuracy^*a*^	Minor error^*b*^	Very major error^*c*^	Categorical agreement
*S. aureus* (13,867/13,510)	99.98%	0.02%	0.00%	97.71%
MRSA (5,906/5,549)	99.95%	0.05%	0.00%	94.63%
MSSA (7,961/7,961)^*d*^	100.00%	0.00%	0.00%	100.00%
*S. pneumoniae* (3,245/3,245)	99.48%	0.46%	0.06%	99.48%
β-hemolytic streptococci (1,768/1,768)^*d*^	100.00%	0.00%	0.00%	100.00%
*Haemophilus* spp. (2,278/2,273)	98.86%	0.84%	0.31%	98.81%
Enterobacterales (21,205/15,979)	98.73%	0.87%	0.40%	89.25%
*E. coli* (9,297/7,301)	99.97%	0.03%	0.00%	94.65%
*K. pneumoniae* (4,054/3,224)	99.91%	0.06%	0.03%	96.47%

^*a*^ Number of isolates susceptible to both ceftobiprole and ceftaroline divided by the total number of ceftaroline-susceptible isolates.

^*b*^ Number of ceftobiprole-intermediate divided by the number of ceftaroline-susceptible isolates.

^*c*^ Number of ceftobiprole-resistant divided by the number of ceftaroline-susceptible isolates.

^*d*^ All isolates were susceptible to both ceftaroline and ceftobiprole.

^*e*^ MRSA, methicillin-resistant *S. aureus*; MSSA, methicillin-susceptible* S. aureus*.

All MSSA isolates (*n* = 7,961) were susceptible to both ceftobiprole and ceftaroline; thus, the categorical agreement was 100.0% and the predictable ceftobiprole susceptibility rate was 100.0% for MSSA isolates susceptible to ceftaroline (Table 3; Fig. S1). When tested against MRSA (*n* = 5,906), the total categorical agreement between ceftobiprole and ceftaroline MIC results was 94.63%. The predictable ceftobiprole susceptibility rate was 99.95% for MRSA isolates susceptible to ceftaroline, the minor error rate was only 0.05%, and no very major errors were observed (Table 3; Fig. S2).

**Fig 2:**
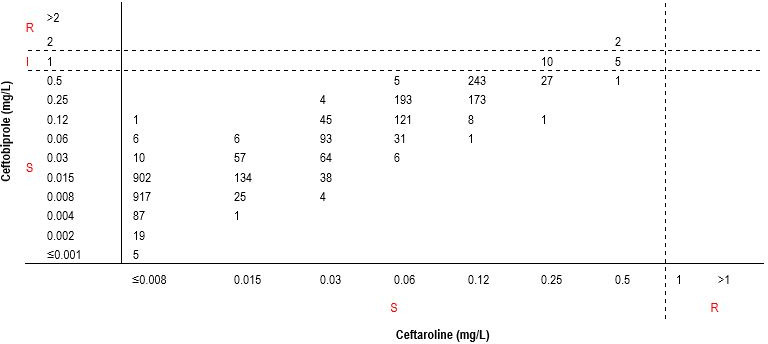
Correlation between ceftobiprole and ceftaroline MIC values for *Streptococcus pneumoniae* (*n* = 3,245)

All *S. pneumoniae* isolates (*n* = 3,245) were susceptible to ceftaroline, and 3,228 of those isolates (99.48%) were susceptible to ceftobiprole. The categorical agreement and the predictable ceftobiprole susceptibility rate both were 99.48% for *S. pneumoniae* isolates susceptible to ceftaroline. Minor error and very major error rates were 0.46% (15/3,228) and 0.06% (2/3,228), respectively (Table 3; Fig. 2). All β-hemolytic streptococci (*n* = 1,768) were susceptible to both ceftobiprole and ceftaroline; thus, the categorical agreement was 100.0% and the predictable ceftobiprole susceptibility rate was 100.0% for β-hemolytic streptococci susceptible to ceftaroline (Table 3).

**Fig 3:**
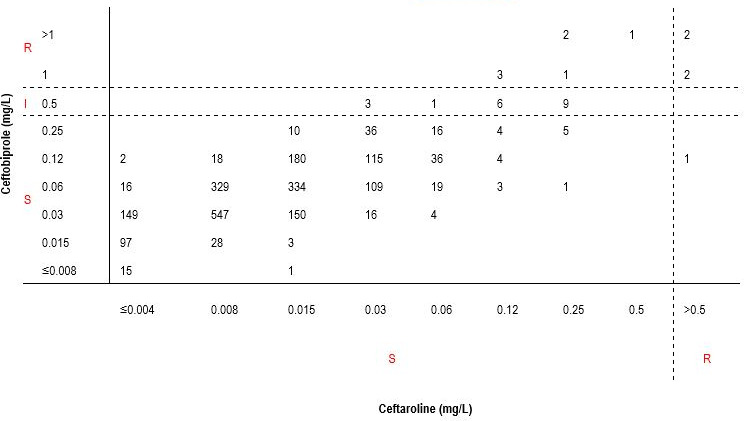
Correlation between ceftobiprole and ceftaroline MIC values for *Haemophilus* spp. (*n* = 2,278)

The total categorical agreement between ceftobiprole and ceftaroline MIC results for *Haemophilus* spp. was 98.81%. Among 2,273 *Haemophilus* spp. isolates susceptible to ceftaroline, 2,247 (98.86%) were also susceptible to ceftobiprole. Therefore, the predictable ceftobiprole susceptibility rate was 98.86% (2,247/2,273) for *Haemophilus* spp. isolates susceptible to ceftaroline. The minor error rate was 0.84% (19/2,273), and the very major error rate was 0.31% (7/2,273; Table 3; Fig. 3).

**Fig 4:**
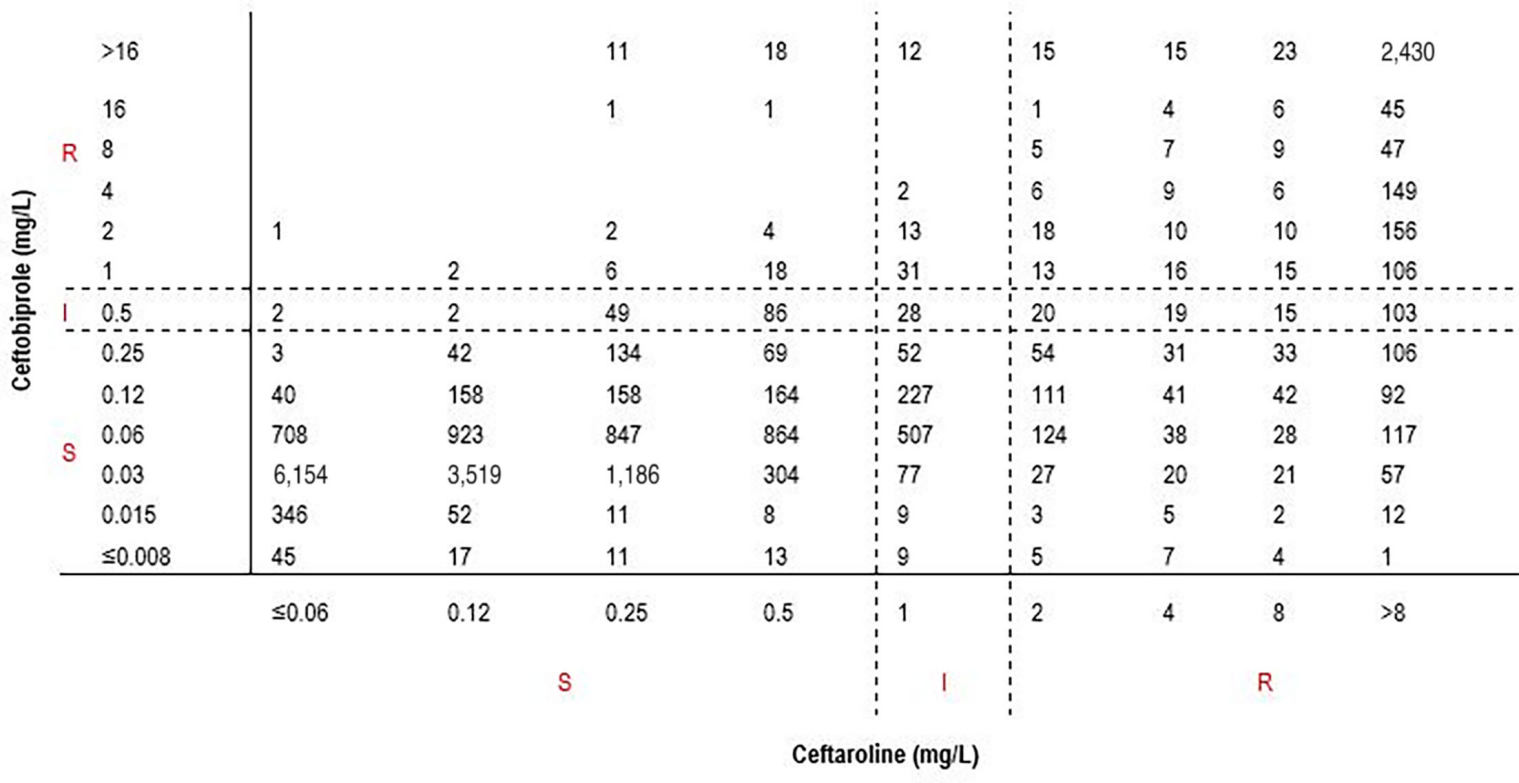
Correlation between ceftobiprole and ceftaroline MIC values for Enterobacterales (all species combined; *n* = 21,205)

The overall categorical agreement for all Enterobacterales species combined (*n* = 21,205) was 89.25%, and susceptibility rate was higher for ceftobiprole (83.2%) compared to ceftaroline (75.4%; Table 2). Among 15,979 Enterobacterales isolates susceptible to ceftaroline, 15,776 (98.73%) were also susceptible to ceftobiprole. Thus, the predictable ceftobiprole susceptibility rate was 98.73% for Enterobacterales isolates susceptible to ceftaroline. The minor error rate was 0.87% (139/15,979) and the very major error rate was 0.40% (64/15,979; Table 3; Fig. 4).

The two Enterobacterales species listed in the ceftobiprole US FDA package insert (4), *Escherichia coli* and *Klebsiella pneumoniae*, were analyzed separately (Tables 1 and 2; Fig. S3 and S4). Among 7,301 *E. coli* isolates susceptible to ceftaroline, 7,299 (99.97%) were also susceptible to ceftobiprole, resulting in a predictable ceftobiprole susceptibility rate of 99.97% for *E. coli* isolates susceptible to ceftaroline. The minor error rate was 0.03% (2/7,301), and there were no very major errors for *E. coli* (Table 3; Fig. S3). When tested against *K. pneumoniae*, 3,224 *K. pneumoniae* isolates were susceptible to ceftaroline, and 3,221 (99.91%) of those isolates were also susceptible to ceftobiprole. Therefore, the predictable ceftobiprole susceptibility rate was 99.91% for *K. pneumoniae* isolates susceptible to ceftaroline. The minor error rate was 0.06% (2/3,224), and the very major error rate was 0.03% (1/3,224; Table 3; Fig. S4).

## DISCUSSION

Surrogate susceptibility tests have been extensively used in clinical practice. These tests usually consist of using susceptibility results from one drug to predict susceptibility to a compound with a similar chemical structure (9–11, 14, 18, 19). For example, the CLSI recommends using cefazolin (a first-generation cephalosporin) MIC value of ≤16 mg/L to predict susceptibility for *E. coli*, *K. pneumoniae*, and *Proteus mirabilis* isolates from uncomplicated urinary tract infections to seven oral cephalosporins, including the third-generation cephalosporins cefpodoxime and cefdinir (10, 11, 14).

The CLSI provides procedures for the evaluation of antimicrobial susceptibility tests that can be applied to surrogate tests. In addition to providing guidelines for study design and data analysis, the CLSI recommends that when a test is compared to a reference method, the categorical agreement of the test must be ≥90%, the minor error rate must be ≤10%, and the major and very major error rates must be <3% (16, 17). Accordingly, the results of the comparative analyses of ceftobiprole and ceftaroline MIC values for 42,363 clinical bacterial isolates presented here clearly indicate that susceptibility to ceftobiprole can be inferred by susceptibility to ceftaroline with a high precision. The accuracy of the surrogate test (susceptibility to ceftaroline) to predict susceptibility to ceftobiprole was >99% for *S. aureus* (99.98%), including MRSA (99.95%), *S. pneumoniae* (99.48%), β-hemolytic streptococci (100.00%), *E. coli* (99.97%), and *K. pneumoniae* (99.91%). Accuracy was also extremely high for *Haemophilus* spp. (98.86%) and for all Enterobacterales species combined (98.73%). Moreover, minor error rates (categorizing a ceftobiprole-intermediate as a ceftobiprole-susceptible) were <1% for all organism groups evaluated. Very major errors (categorizing a ceftobiprole-resistant as ceftobiprole-susceptible) were observed at very low rates (≤0.40%) and only with *S. pneumoniae* (0.06%), *Haemophilus* spp. (0.31%), Enterobacterales (0.40%), and *K. pneumoniae* (0.03%).

It is important to emphasize that this investigation was specifically designed to evaluate the ability of susceptibility to ceftaroline to predict susceptibility to ceftobiprole. We did not analyze the population of isolates that were not susceptible to ceftaroline. Thus, we cannot derive any correlation between nonsusceptibility to ceftaroline and nonsusceptibility to ceftobiprole, and this correlation probably varies widely among the organism groups evaluated. Moreover, we did not evaluate the ability of susceptibility to ceftobiprole to predict susceptibility to ceftaroline.

The fact that all testing was performed in one laboratory can be considered a limitation of the study. However, it is important to observe that the bacterial isolates came from 34 medical centers located throughout the 9 US Census Divisions and the testing was performed by various technician teams during a 5-year period. Although these limitations should be considered when interpreting the results and conclusions, it is very unlikely that they have introduced important bias to the study.

In conclusion, the surrogate testing strategy outlined here could facilitate rapid clinical use of ceftobiprole in the United States pending the more necessary availability of commercial susceptibility testing devices to direct testing this compound.

## SUPPLEMENTAL MATERIAL

Figures S1 to S4 jcm.01706-25-s0001.docx Scattergrams for MSSA, MRSA, *E. coli*, and *K. pneumoniae*.

ASM does not own the copyrights to Supplemental Material that may be linked to, or accessed through, an article. The authors have granted ASM a non-exclusive, world-wide license to publish the Supplemental Material files. Please contact the corresponding author directly for reuse.
