# Supplementary material for: Inference of ceftobiprole susceptibility through surrogate testing of ceftaroline
Source: J Clin Microbiol. 2026 Feb 24;64(4):e01706-25. doi: 10.1128/jcm.01706-25 (PMC13059735; doi:10.1128/jcm.01706-25)
Supplement: Figures S1 to S4 — Scattergrams for MSSA, MRSA, E. coli, and K. pneumoniae. [file jcm.01706-25-s0001.docx]

Figure S1 Correlation between ceftobiprole and ceftaroline MIC values for methicillin-susceptible Staphylococcus aureus (n=7,961)

| **Ceftobiprole (mg/L)** | R | >4 |  |  |  |  |  |  |  |  |  |
| --- | --- | --- | --- | --- | --- | --- | --- | --- | --- | --- | --- |
|  | I | 4 |  |  |  |  |  |  |  |  |  |
|  | S | 2 |  |  |  |  |  |  |  |  |  |
|  |  | 1 |  |  | 7 | 13 |  |  |  |  |  |
|  |  | 0.5 | 4 | 267 | 5041 | 348 |  |  |  |  |  |
|  |  | 0.25 | 37 | 793 | 1386 |  |  |  |  |  |  |
|  |  | 0.12 | 23 | 31 | 4 |  |  |  |  |  |  |
|  |  | 0.06 | 5 | 1 |  |  |  |  |  |  |  |
|  |  | ≤0.03 | 1 |  |  |  |  |  |  |  |  |
|  |  |  | ≤0.06 | 0.12 | 0.25 | 0.5 | 1 | 2 | 4 | 8 | >8 |
|  |  |  | S | | | | | I | | R | |
|  |  |  | **Ceftaroline (mg/L)** | | | | | | | | |

Figure S2 Correlation between ceftobiprole and ceftaroline MIC values for methicillin-resistant Staphylococcus aureus (n=5,906)

| **Ceftobiprole (mg/L)** | R | >4 |  |  |  |  |  |  |  |  |  |
| --- | --- | --- | --- | --- | --- | --- | --- | --- | --- | --- | --- |
|  | I | 4 |  |  |  |  | 3 | 43 |  |  |  |
|  | S | 2 |  |  |  | 51 | 1197 | 309 | 1 |  |  |
|  |  | 1 |  |  | 11 | 2279 | 1680 | 4 |  |  |  |
|  |  | 0.5 |  | 4 | 92 | 209 | 3 |  |  |  |  |
|  |  | 0.25 |  | 1 | 10 | 7 |  |  |  |  |  |
|  |  | 0.12 |  | 1 | 1 |  |  |  |  |  |  |
|  |  | 0.06 |  |  |  |  |  |  |  |  |  |
|  |  | ≤0.03 |  |  |  |  |  |  |  |  |  |
|  |  |  | ≤0.06 | 0.12 | 0.25 | 0.5 | 1 | 2 | 4 | 8 | >8 |
|  |  |  | S | | | | | I | | R | |
|  |  |  | **Ceftaroline (mg/L)** | | | | | | | | |

Figure S3 Correlation between ceftobiprole and ceftaroline MIC values for *Escherichia coli* (*n*=9,297)

| **Ceftobiprole (mg/L)** | R | >16 |  |  |  |  |  |  | 1 | 1 | 1432 |
| --- | --- | --- | --- | --- | --- | --- | --- | --- | --- | --- | --- |
|  |  | 16 |  |  |  |  |  |  | 1 |  | 11 |
|  |  | 8 |  |  |  |  |  | 1 | 2 | 1 | 5 |
|  |  | 4 |  |  |  |  |  | 1 | 2 | 1 | 9 |
|  |  | 2 |  |  |  |  |  | 2 |  |  | 7 |
|  |  | 1 |  |  |  |  | 1 | 1 | 2 | 4 | 13 |
|  | I | 0.5 |  |  | 2 |  | 4 | 2 | 5 | 4 | 40 |
|  | S | 0.25 | 1 | 3 | 5 | 5 | 14 | 15 | 6 | 17 | 41 |
|  |  | 0.12 | 6 | 13 | 22 | 38 | 46 | 34 | 15 | 21 | 11 |
|  |  | 0.06 | 374 | 379 | 363 | 211 | 144 | 40 | 9 | 2 | 4 |
|  |  | 0.03 | 3615 | 1538 | 479 | 106 | 14 | 5 | 2 | 1 | 2 |
|  |  | 0.015 | 130 | 7 | 1 | 1 |  |  |  |  |  |
|  |  | ≤0.008 | 2 |  |  |  |  |  |  |  |  |
|  |  |  | ≤0.06 | 0.12 | 0.25 | 0.5 | 1 | 2 | 4 | 8 | >8 |
|  |  |  | S | | | | I | R | | | |
|  |  |  | **Ceftaroline (mg/L)** | | | | | | | | |

Figure S4 Correlation between ceftobiprole and ceftaroline MIC values for *Klebsiella pneumoniae* (*n*=4,054)

| **Ceftobiprole (mg/L)** | R | >16 |  |  |  |  |  |  | 2 | 6 | 592 |
| --- | --- | --- | --- | --- | --- | --- | --- | --- | --- | --- | --- |
|  |  | 16 |  |  |  |  |  |  | 1 | 4 | 6 |
|  |  | 8 |  |  |  |  |  | 2 | 2 | 2 | 4 |
|  |  | 4 |  |  |  |  |  | 2 | 1 | 1 | 11 |
|  |  | 2 |  |  |  |  | 2 | 9 | 5 | 6 | 10 |
|  |  | 1 |  |  |  | 1 | 7 | 2 | 5 | 6 | 5 |
|  | I | 0.5 |  |  | 1 | 1 | 6 | 7 | 6 |  | 2 |
|  | S | 0.25 |  |  | 1 | 6 | 11 | 11 | 6 |  | 1 |
|  |  | 0.12 | 10 | 16 | 20 | 30 | 18 | 6 | 3 |  |  |
|  |  | 0.06 | 101 | 178 | 136 | 131 | 31 | 7 | 5 |  | 2 |
|  |  | 0.03 | 1247 | 985 | 206 | 42 | 4 | 4 | 2 | 3 | 2 |
|  |  | 0.015 | 91 | 19 |  |  |  |  |  |  |  |
|  |  | ≤0.008 | 2 |  |  |  |  |  |  |  |  |
|  |  |  | ≤0.06 | 0.12 | 0.25 | 0.5 | 1 | 2 | 4 | 8 | >8 |
|  |  |  | S | | | | I | R | | | |
|  |  |  | **Ceftaroline (mg/L)** | | | | | | | | |
